# Spontaneous Intracranial Hemorrhages in a Community of Asian Americans: Case Series and Literature Review

**DOI:** 10.7759/cureus.52128

**Published:** 2024-01-11

**Authors:** Henry Querfurth, Izabella Marczak, Nasrin Rahimian, Amir Jijakli, Deborah Green-LaRoche

**Affiliations:** 1 Neurology, Tufts Medical Center, Boston, USA

**Keywords:** s: biomarkers, case-series, incidence and prevalence, asian americans, spontaneous intracranial hemorrhage

## Abstract

Background and objectives

Several Asian populations abroad are reported to have a higher prevalence of spontaneous intracranial hemorrhages (sICH) and a greater proportion of all stroke types attributed to ICH compared to non-Asians. However, the causes are unknown, and few studies have examined the issue among Asian Americans. This report aims to highlight some less common, but not rare, clinical features that could bear on several pathophysiological factors, by presenting a selected case series of 13 Asian American patients admitted to a Boston-based healthcare system and hospital.

Methods

The selected cases were classified into six categories based on presumed sICH mechanisms including vasculopathy, hypertensive crises, moyamoya disease/syndrome, venous sinus thrombosis, brainstem hemorrhages, and arterial malformation/aneurysm. We also examined 5 years of medical records at our institution, a single healthcare system among several in a large urban area having its main hospital embedded in an Asian community, to arrive at stroke-type proportions, comparing our Asian to non-Asian population. ICH cases excluded trauma, coagulopathy, and hemorrhagic transformation. ICH patient counts were compared to acute ischemic stroke and subarachnoid hemorrhage across various ethnicities.

Results

Pathophysiology-biomarker correlations within each ICH stroke category were reviewed, some possibly having specificity for Asian populations. We found some evidence to support an increased proportion of sICH among all stroke types in our Asian American patients, relative to other ethnic groups. A higher apparent estimate of sICH incidence in Asian Americans vs. Caucasians was also uncovered. However, these did not reach statistical significance and so no conclusion on risk could be made from this preliminary study.

Conclusions

We review the extensive literature on epidemiology and genetic markers and affirm that an awareness of the potential increased risk of sICH in this expanding population is clinically prudent. An expanded epidemiologic study to refine ICH risk estimates in Asian Americans is planned.

## Introduction

The prevalence of intracranial hemorrhage (ICH) and the proportion of all strokes due to ICH versus acute ischemic stroke (AIS) is reported to be increased among Asians relative to Caucasians [1-4]. For instance, the age- and sex-adjusted yearly incidence of ischemic stroke (IS) is 43% lower in Chinese than Caucasians, whereas ICH was 18% higher [4]

Among overall strokes, a higher prevalence of ICH exists in Chinese (~33%) versus Caucasian (~12%) populations [1,2]. The incidence of sICH is 51.8/100K in Asians, twice that in Caucasians (24.2/100K)[3]. sICH cases among Chinese had a mean age of 58, most with underlying hypertension (HTN) and having deep cerebral bleeds. However, younger patients (<44) tended to harbor a vascular anomaly whereas older patients (>75) had a higher occurrence of lobar ICH [5]. A meta-analysis found that isolated HTN was a greater risk for ICH in Chinese than in Caucasians (Odds Ratio (OR) 7.2 vs. 3.1)[6].

From antithrombotic trials, East Asians have a higher risk of hemorrhagic stroke compared to Caucasians, referred to as the ‘East Asian paradox’ [7]. Asian patients receiving recombinant tissue plasminogen activator (rtPA) for AIS have an increased risk of hemorrhagic complications relative to Caucasian patients (OR =1.36 to 1.51) [8,9]. The risk for ICH is higher yet on warfarin for atrial fibrillation (AF) relative to whites (Hazards Ratio (HR)=4.0) [10]. However, not all studies can confirm this [11]. The pathological, genetic, and acquired mechanisms behind these observations are unknown and likely multifactorial. Alcohol and hypertension are particularly major risks for ICH over AIS in the Chinese [12]. Although substantial literature exists on ICH in Asians, there is less documentation in the Asian American communities. In a single study, the risk of hemorrhagic stroke in Asian Americans was increased relative to whites (RR 1.6)[13]. An analysis of primary ICH numbers in Asian Americans on a US national scale appeared in a recent research letter [14]. Most of their 40,582 retrospective cases over 15 years were in the Pacific division of the US. They found a higher proportion of sICH among all stroke types in Asian Americans (18.9% vs. 11.1% non-Hispanic whites; OR=1.88). Interestingly, the incidence rates were not different. Their demographic review found higher cardiovascular risk burden, more favorable economic indices, lower age at first sICH, and severer outcome in the aggregate US-Asian population [14]. There is scant epidemiologic data on either Asian-Europeans or Eurasians.

We present a selected case series of primary intracranial hemorrhages, grouped by mechanism, in a dense non-affluent Asian American community served by a Boston-based healthcare system and hospital. Uncommon circumstances are highlighted that relate to ongoing research into pathophysiological factors unique to this population. These also raise awareness of certain risk factors for ICH to watch for in this population.

## Materials and methods

A sample of 13 cases from over the last five years was selected to highlight both common and less common, but not rare, circumstances contributing to an increased risk for spontaneous ICH in an Asian American community. These were chosen empirically only to represent the most common major pathophysiologic mechanisms of ICH that we encountered. Non-primary brain hemorrhage cases were excluded including: undertreated essential hypertension, anticoagulant use (warfarin, rtPA or non-vitamin K antagonist oral anticoagulants (NOAC)), known blood dyscrasia, fibrinolytic hemorrhagic complications of AIS, thrombectomy, head trauma, isolated epi/subdural hematomas, hemorrhagic conversion of acute ischemic strokes, and atrial fibrillation. No case had concurrent COVID-19 infection.

In order to derive proportions of stroke type across a local community of Asian Americans, medical records were reviewed from Jan 2017 to Dec 2021 involving an all-stroke cases search among the Asian-American population admitted over the five years to either the Neurology or Neurosurgery service or respective ICUs. 350 records were retrieved and reviewed. Asian Americans include ancestry from far eastern and southeast countries as well as the Indian subcontinent. These were compared to all-stroke cases involving Caucasians (n=1078), African American/Blacks (n=152), and ‘Other’ (n=116) referring to Hispanic, Native American/Alaskan, and unknown ethnic groups. Most Asian American cases, especially those of age 49 and over, were likely born abroad. The younger cases were English speaking and probably born and/or educated in the USA. The strokes were divided into Acute Ischemic Stroke (AIS), Primary Intracranial Hemorrhage (ICH), and Primary Subarachnoid Hemorrhage (SAH) groups. From the cases' numbers, the percentages of each type of stroke across ethnicity and percentages (or proportion) of stroke types within each ethnic background were computed. We also estimated an apparent incidence (no./100K/yr) of AIS, sICH, and SAH for each ethnic grouping by dividing the number of strokes of a given type admitted to one Boston institution embedded in a large Asian community (Tufts Medical Center) over 5 yrs., by the whole adult population in Boston over age 18 within each ethnic group. Population numbers by race were obtained from the 2020 U.S.A. Census Bureau, Population Estimates Program (PEP), American Community Survey (www.data.census.gov/table?q=Boston+city,+Massachusetts)

Statistics on Asian vs. Caucasian (or vs. all combined non-Asian) groupings with respect to either each stroke category (AIS, sICH, SAH) or just sICH vs. combined non-ICH) were performed on patient counts, as well as on estimated apparent incidences, using standard Chi-Square test (X2), in 2x3 and 2x2 table formats, respectively (GraphPad.com). Bonferroni correction of significance values for multiple comparisons was not made due to a non-applicable Type 1 error and a low number of comparisons.

In reporting the following cases and analysis of stroke proportion at our institution and use of population data to arrive at an estimate of incidence, we followed the PROCESS reporting guidelines for case series [15] and STROBE guidelines for cross-sectional study design where applicable [16].

Study approval and permissions were granted by the Institutional Review Board of the Tufts Medical Center under study number MOD-01-00000734. Under institutional guidelines, it was not required to obtain individual consents.

## Results

Case 1

64-year-old Chinese woman presented to the emergency department (ED) with a severe occipital headache exacerbated by coughing starting nine days prior. Admission vitals, general neurological examination and laboratory workup were unremarkable. Computed tomography (CT) of the head revealed a small focus of acute subarachnoid hemorrhage (SAH) along the right posterior temporal lobe. CTA showed distal middle cerebral artery (MCA) beading concerning for vasculitis or reversible cerebral vasoconstriction syndrome (RCVS). MRI confirmed SAH and MR angiogram (MRA) redemonstrated mild to moderate vessel wall irregularities along the bilateral anterior cerebral artery (ACA) and posterior cerebral arteries (PCA). Elevated blood pressure was treated with verapamil which also improved the headaches. Vasculitis and infectious panels were unremarkable except for low complement C3 and evidence for previous hepatitis B infection. Shortly after a 2 day admission she was noted in the outpatient clinic to have developed a scaly, erythematous rash on the dorsal fingers and two aphthous ulcers in her mouth. A repeat MRI with MRA and rheumatology consultation were scheduled but the patient was lost to follow up.

Case 2

85-year-old Chinese male with diabetes mellitus type 2, hypertension, hyperlipidemia, and tobacco use was brought to the ED after one week of altered mental status, agitation and aggressiveness. According to family, he had become mute after a period of speaking only in simple phrases and lost the ability to care for himself. Four months previous to this, he had a progressive speech problem that resolved. On admission he was hypertensive (192/80 mmHg), tachycardic and oriented to person but not time or place. He struggled to complete nominally complex tasks. Recent memory was impaired. Initial head CT revealed an acute/subacute intraparenchymal hemorrhage (IPH) along the medial right temporal lobe that measuring 2.4 cm with local mass effect and a focus of SAH overlying the superior left frontal lobe. The lumbar puncture (LP) viral panel returned negative and acyclovir was stopped. CTA of the head showed multifocal, multivessel stenoses, including a critical focal stenosis in the left MCA-M1 segment. There was asymmetrically diminished collateral flow into the left MCA territory. MRI of the head showed expected evolutions of the IPH and SAH. His hospitalization was complicated by agitation and after eight days he became less responsive. Repeated CT showed a new large ischemic stroke in the left frontal and temporal lobe, insular cortex, and parietal cortex. The patient’s family decided on discharge home with hospice care.

Case 3

42-year-old Cambodian male was transferred from another hospital to manage an acute IPH and SAH. The night before admission, he developed sudden right-sided weakness and language deficits and fell. In the ED, he was hypertensive (200/120s mmHg). Past medical history consisted of untreated hypertension and cocaine use decades earlier. Glasgow Coma Scale (GCS) score was 7 and he was intubated. Laboratory studies showed an elevated creatinine level, microcytic anemia, and hypokalemia. CT head showed an intraparenchymal hematoma (IPH) measuring 3.6x3.8x3.8 cm in the left basal ganglia and insula, a small SAH in the left Sylvian fissure and interpeduncular cistern and local mass effect (3 mm rightward midline shift). MRA showed no evidence of aneurysm or arteriovenous malformation (AVM). He required nicardipine, enalaprilat, labetalol, and hydralazine for blood pressure management. Later, he was transitioned to lisinopril, amlodipine, and labetalol. The patient had a fever with abdominal discomfort and increasing level of creatinine. CT of the abdomen and pelvis revealed innumerable bilateral renal and hepatic cysts consistent with autosomal dominant polycystic kidney disease (ADPKD). His clinical condition progressively improved. At the time of discharge for rehabilitation, he was aphasic and hemiparetic.

Case 4

59-year-old Chinese male with a 10-year smoking history and untreated hypertension presented with two days of gradual onset headache, blurred vision, weakness, nausea/vomiting, and instability with falls. On admission, he was hypertensive (237/155 mm/Hg) and started on Lisinopril. Neurological exam showed rightward nystagmus, mild ataxia on right finger-to-nose, and drift of left upper extremity. CT of the head showed right cerebellar hyperdensity measuring 1.4 cm, suspected to be IPH. There was effacement of the fourth ventricle with surrounding edema out of proportion to the hemorrhage size. An atypical presentation of posterior reversible encephalopathy syndrome (PRES) was suspected. Brain MRI showed right cerebellar hemorrhage, in addition to a left frontal subcortical hemorrhage and punctate hemorrhages in the pons and midbrain. MRA showed no abnormalities. Blood pressures stabilized on amlodipine and carvedilol. His condition gradually improved and he was discharged home.

Case 5

**Figure 1:**
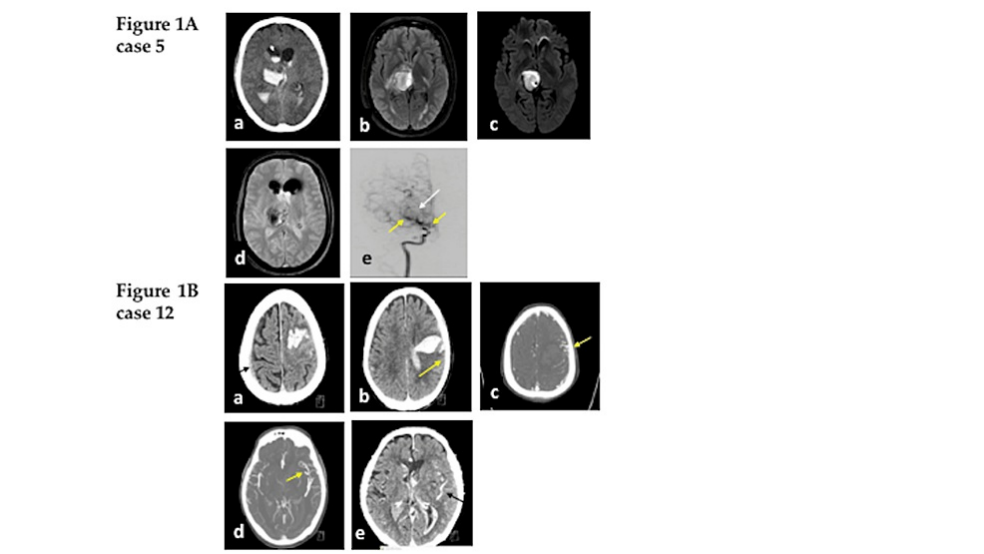
Multicompartment (ICH, IVH, SAH) intracranial hemorrhage examples in an Asian American population. (1A) Case 5: Axial non-contrast head CT (a) shows acute right thalamic parenchymal hemorrhage with pan-ventricle and tentorial subarachnoid extension. Axial MRI *FLAIR* (b) *DWI* (c) and gradient echo T2(*) (d) show acute right thalamic parenchymal hemorrhage. CT angiography of RICA (e) demonstrating severe stenosis of *RICA* (yellow arrow) with the pronounced appearance of lenticulostriate vessels (white arrow). (1B) Case 12: Axial non-contrast head CT (a) shows high left frontoparietal ICH with associated small volume subarachnoid hemorrhage (SAH) (yellow arrow) and right frontoparietal subdural hematoma (SDH)(black arrow). Axial non-contrast CT (b) shows more caudal large left parietal ICH with IVH.  CT angiography shows high parietal (c) and lower down (d) AVM measuring 2.6 cm in the largest dimension (yellow arrow). CT angiography (e) delayed phase shows venous drainage pattern (black arrow). AVM - arteriovenous malformation), FLAIR - fluid-attenuated inversion recovery, DWI - diffusion-weighted imaging, RICA - right internal carotid artery, ICH - intracerebral hemorrhage, IVH - intraventricular hemorrhage, SAH - subarachnoid hemorrhage.

50-year-old Chinese female was admitted to the ICU for acute left-sided numbness, paresthesia, weakness and somnolence followed by loss of consciousness. Three months prior she was evaluated for bilateral hand numbness and weakness, awakening her in the mornings. These would remit during the daytime. A wrist splint and gabapentin did not help. Electromyography/nerve conduction velocity (EMG/NCV) studies were normal without evidence for carpal tunnel syndrome (CTS). On arrival at the ED, she was unresponsive with a Glasgow coma scale of 3 and required intubation. She had a history of untreated hypercholesterolemia. Initial neurological examination was noted for a dilated right pupil, absent corneal reflex on the left side, left facial droop and left Babinski sign. Laboratory investigations uncovered hypokalemia and hyperglycemia. CT of the head showed an acute right thalamic IPH with pan-ventricle and tentorial subarachnoid extension and prominence of lateral ventricle sizes suggestive of early hydrocephalus (Fig. 1A). The hematoma and associated edema caused a 4 mm right to left midline shift and medialization of the right temporal lobe indicating early uncal herniation. An external ventricular drain (EVD) was placed. A head CTA showed severe stenosis of bilateral Right > Left MCA with the pronounced appearance of lenticulostriate vessels suspicious for Moyamoya disease (Figure 1A-e). After nine days, she deteriorated and could no longer move her right arm. Repeat CT scan showed a new small 8 mm contralateral (left) periventricular hemorrhage surrounding the posterior limb of the internal capsule. During the prolonged hospitalization, she underwent tracheostomy and PEG placement. The EVD was replaced by an endoscopic third ventriculostomy (ETV). She was discharged to a rehabilitation facility. When readmitted for fevers after four days, a head CT showed increased size of the left corona radiata (CR) hemorrhage and lateral ventricles. After three more days, worsening ventriculomegaly prompted a flow study that showed occlusion of the ETV. She underwent a third ventriculostomy. Her condition gradually improved. She was discharged to a long-term facility but hospitalized multiple times since with new ICHs and complications, including a pulseless electrical activity (PEA) arrest.

Case 6

49-year-old Chinese male was transferred from another hospital for management of an intraventricular hemorrhage (IVH), initially presenting after sudden onset of severe headache, vomiting, and transient loss of consciousness. An active smoker, he had a history of hypertension and a prior ICH of unknown etiology. Admission vitals were normal but he was oriented only to the person. Laboratory investigation showed leukocytosis. CT of the head showed extensive pan-intraventricular hemorrhage with a moderate-size SAH in perimesencephalic and interpeduncular cisterns. He underwent EVD placement for hydrocephalus prevention. A head CTA with contrast showed abnormal appearances of the bilateral internal carotid artery terminus, completely absent on the right and small on the left, with absent proximal right A1, bilateral M1 segments, and small left A1 segment. There were collateral blood vessels seen around the internal carotid artery terminus and along the Circle of Willis. Findings were suggestive of moyamoya disease. He was treated with intraventricular recombinant tissue plasminogen activator (rtPA)that aided the resolution of his IVH. During hospitalization, he developed the syndrome of inappropriate antidiuretic hormone secretion (SIADH), treated with salt tabs and fluid restriction. After gradual improvement, he was discharged to a rehabilitation facility.

Case 7

20-year-old Asian American female was transferred from another hospital to manage bilateral frontal subdural hematomas (SDH). Five days before admission, she complained of headaches and gradually became disoriented. She had a past medical history of immune thrombocytopenia (ITP), congenital methemoglobinemia, and a cryptogenic ischemic stroke two years ago. Her medications included daily eltrombopag (Promacta -** **Novartis Pharma AG, Basel, Switzerland) and aspirin. On arrival, she was hypotensive and her GCS was 6. Her initial laboratory exam showed thrombocytopenia that required a platelet transfusion. MRI and magnetic resonance venogram​​​​​​​ (MRV) revealed an absence of flow-related enhancements with heterogeneous intrinsic T1 hyperintense signal within the superior, straight, transverse, and sigmoid sinuses concerning for extensive dural venous sinus thrombosis. There were multiple foci of acute IPHs with the largest area in the left temporal lobe and a small focus in the left occipital lobe, bilateral frontal SDH, scattered bilateral SAH and acute ischemic changes within the left temporal and occipital lobes. The patient underwent Penumbra suction thrombectomy (Penumbra Inc., Alameda, USA​​​), Trevo stent-assisted mechanical thrombectomyx 2 (Stryker, Kalamazoo, USA), and Solitaire stent-assisted mechanical thrombectomy (Medtronic, Minneapolis, USA), with catheter-delivered rtPA to the venous sinuses. After the last procedure, she became unresponsive. Repeated head CT scan revealed progression of the patient's presumed venous infarcts, with a new focus of hemorrhage within the left parietal lobe and extensive edema involving the left hemisphere with a rightward midline shift of 11 mm. She underwent a left hemicraniectomy and a partial left temporal lobectomy. Her level of consciousness (LOC) did not improve, and she expired.

Case 8

24-year-old Chinese woman presented with a gradual onset left headache and decreased LOC. Six months before the incident, her oral contraceptive pill was changed to the higher dose estrogen-containing Sprintec (Sprintec28 (norgestimate and ethinyl estradiol), TEVA Pharmaceuticals, Parsippany, USA)​​​​​​​** **as she was not experiencing periods. The neurological examination revealed bilateral papilledema and non-fluent aphasia. A left hemorrhagic stroke was seen on head CT. The contrast-enhanced sequences showed a large filling defect in the junction between the left transverse and sigmoid sinus, indicating cerebral venous sinus thrombosis (CVST). Warfarin was started and continued for six months. Her expressive aphasia dramatically improved with speech therapy over the next few weeks. Investigations for thrombophilia and genetic abnormalities found her heterozygous for the plasminogen activator inhibitor-1 4G/5G genotype. Due to the moderate risk of hypercoagulability, daily aspirin was started. One year after the incident, she had a nocturnal seizure which was well controlled thereafter with oxcarbazepine. 

Case 9

59-year-old Vietnamese man with diet-controlled diabetes mellitus and hyperlipidemia presented to the emergency department having acute onset right facial and upper extremity numbness and unsteady gait. The patient was a chronic smoker. Vital signs were normal. Initial neurological examination was unremarkable except for decreased sensation on the right side and impaired heel-to-shin test on the right lower extremity. Brain CT scan revealed 1.3 x 0.8 x 0.9 cm left midbrain IPH. CTA showed a poorly defined enhancement consistent with a venous anomaly. The focal, circumscribed area of hyperattenuation in the left aspect of the midbrain did not have significant surrounding edema or local mass effect, thus suggesting a vascular malformation such as capillary telangiectasia or cavernous malformation. Decreased pinprick sensation on the right side and unsteady gait persisted after discharge.

Case 10

51-year-old Asian American male presented with acute worsening of baseline left hemiparesis and dysarthria. He had a past medical history of HTN, diabetes mellitus, and pontine hemorrhagic stroke 5 years prior. Admission blood pressure was 162/106 mmHg. His LOC declined rapidly during the first few hours of admission. CT scan revealed an acute IPH centered within the ventral pons, measuring 2.1 cm x 3.1 cm x 2.5 cm, with surrounding edema resulting in a mild mass effect on the adjacent parenchyma and effacement of the fourth ventricle. The bleeding was at the same anatomical location as the previous IPH. He developed Locked-in Syndrome and could not follow commands except for minimal vertical eye movements and blinking. He was managed with anti-hypertensives. The family requested extubation and comfort measures only (CMO).

Case 11

57-year-old Chinese male with unknown medical history was brought to the ED with acute headaches followed by rapid unresponsiveness. Two weeks before the incident, he suffered a mild head strike without clinical sequelae, bleeding or swelling. Vitals were stable and GCS was 5. His initial neurological examination showed anisocoria with a left fixed and dilated pupil and left hemiplegia. A brain CT scan revealed an extensive IVH involving all four ventricles and a right parafalcine frontal lobe infarct. Digital subtraction angiography​​​​​​​ (DSA)showed a small distal right posterior cerebral artery (PCA) arteriovenous malformation (AVM) with a nidal aneurysm. After placing an EVD in the right frontal ventricle, consciousness improved and anisocoria resolved. Subsequent imaging revealed the development of an inferior left frontal/caudate nucleus infarct. CTA showed multifocal vascular irregularities involving the middle, posterior, and anterior cerebral arteries. The IVH was associated with an 8 mm leftward midline shift. Clogging of EVDs was often followed by episodes of right pupil enlargement and worsening mental status secondary to brain herniation. The patient required repeated EVD placements. Intrathecal rtPA was withheld due to the risk of rebleed. Finally, he underwent a septum pellucidotomy, after which his ventricles normalized and symptoms improved.

Case 12

93-year-old Asian male with a history of HTN and dyslipidemia was found unresponsive with left gaze deviation and right hemiparesis, no convulsive movements were reported. On admission, GCS was 10. Vital signs were noted for severe hypertension (230/110 mmHg) and atrial fibrillation (HR ~80). He was treated with labetalol. Head CT showed a large volume left IPH with IVH extension and associated small SAH (Figure 1B). There was a 0.4 cm right frontoparietal SDH, likely subacute (Figure 1B). A suspected Duret hemorrhage in the pons indicating rapid expansion of ICH and downward herniation of the brainstem was reported. An AVM along the lateral left precentral gyrus, measuring 2.6 cm in the largest dimension, was discovered (Figure 1B-c,d). It appeared to have superficial drainage and a supply from the left MCA. He was started on clevidipine drip, hypertonic 3% saline 250 mL, and intubated for airway protection. Given the size and location of the hemorrhage, surgical intervention was deemed unlikely to provide a meaningful benefit. Due to an advance directive, he was made Do Not Resuscitate/Do Not Intubate (DNR/DNI), transitioned to CMO, and expired three days after admission. 

Case 13

67-year-old Chinese male and former smoker with a past medical history of brain lymphoma, hepatitis B, and atrioventricular reciprocating tachycardia (AVRT) was brought to the ED with acute headache and neck pain followed by unresponsiveness. According to the family, he was complaining of mild headache the day before hospitalization. He was admitted in hypertensive crisis (systolic blood pressure 270 mmHg). The neurological examination revealed fixed, unresponsive eyes with anisocoria (Left > Right), absent corneal reflex and no movement of extremities to noxious stimuli. On the way to CT scan, he had two pulseless electrical activity (PEA) cardiac arrests with return of spontaneous circulation. He was intubated and started on pressors. CT and MRI of the head showed a multi-compartmental hemorrhage including marked diffuse SAH, bilateral supratentorial and infratentorial subdural hematomas, and pan-intraventricular hemorrhage. Bilateral infarction of cerebellar hemispheres and vermis with brainstem mass effect and smaller strokes in the basal ganglia were noted. CTA showed a distal V4 segment of the left vertebral artery aneurysm measuring up to 6 mm. An emergent right frontal EVD was placed. The patient was transferred to the ICU. Due to a poor prognosis for functional recovery, family members opted to terminally extubate the patient.

The cases are summarized and grouped by pathophysiologic mechanism in Table 1.

**Table 1:** Selected cases: arranged by etiological categories Abbreviations: AF - atrial fibrillation, AMS - altered mental status, AVM - arteriovenous malformation, CVST - cerebral venous sinus thrombosis, DM - diabetes mellitus, HA - headache, HLD - hyperlipidemia, HTN - hypertension, ICH - intracranial hemorrhage, IPH - intraparenchymal hemorrhage, IVH - intraventricular hemorrhage, ITP - immune thrombocytopenic purpura, LOC - loss of consciousness, OC-oral contraceptive, PCKD - polycystic kidney disease, PRES - posterior reversible leukoencephalopathy syndrome, SAH - subarachnoid hemorrhage, SDH - subdural hemorrhage, RCVS - reversible cerebral vasoconstriction syndrome, HTN hx - hypertension history + denotes if hypertension was present historically and/or present on admission, - denotes its absence.

Case	Age	Type of hemorrhage	Sex	BMI	HTN hx	HTN present	Past medical history	Presenting symptoms	Causation
Vasculopathy									
1	64	SAH	F	23.98	-	+	occasional HA	sudden onset of severe HA	RCVS, PRES
2	85	IPH and SAH	M	20.36	+	+	DM2, HLD, smoking	aphasia, AMS	atherosclerosis
Hypertensive Arteriopathy									
3	42	IPH and SAH	M	unknown	+	++	-	aphasia, dysarthria, right-sided hemiplegia	severe HTN, PCKD
4	59	IPH	M	19.38	+	++	smoking	HA, vomiting, blurred vision	PRES, severe HTN
Moya Moya Disease									
5	50	IPH	F	21.21	-	-	Hypercholesterolemia, Moya Moya disease	left-sided numbness and weakness, LOC	Moyamoya- arteriopathy, vaso-occlusive disease
6	49	IVH, SAH	M	unknown	+	-	HTN, prior IVH, smoking	HA, vomiting	Moyamoya disease
Venous Thrombosis									
7	20	CVST, secondary multi-compartment hemorrhages	F	25.88	-	-	ITP, Congenital Methemoglobinemia, Ischemic stroke	HA, disorientation	Extensive dural venous thromboses, with secondary hemorrhagic and ischemic strokes
8	24	CVST, secondary ICH	F	unknown	-	-	unknown	HA and decreased level of consciousness	Venous sinus thrombosis, OCs, and plasminogen activation
Brainstem Hemorrhages									
9	59	Midbrain ICH	M	19.6	-	-	DM, Hyperlipidemia, smoking	right-sided numbness and unsteady gait	Vascular (venous) malformation
10	51	Pons ICH, recurrent	M	30.62	+	+	Pons ICH, HTN, DM	Left hemiparesis and dysarthria	HTN
Arterial Malformation									
11	57	Extensive IVH	M	23.5	-	-	unknown	Decreased level of consciousness and anisocoria	AVM
12	93	Extensive multi-compartment hemorrhages	M	-	+	++	HTN, HLD, AF	Decreased level of consciousness, Gaze deviation	HTN, AVM
13	67	Multi-compartment hemorrhages (SAH, IVH, SDH)	M	-	-	++	Lymphoma, smoking	HA, coma	HTN, Vertebral artery aneurysm

Next, we sought evidence that the ‘Asian paradox’, an increase in sICH prevalence among Asians compared to Caucasians documented in a majority of literature arising abroad, might also apply to our Asian American community. Table 2 gives the observed in-patient counts over 5 years corresponding to each stroke category and parsed by four ethnic groupings. The Asian population had the highest prevalence (or proportion %) of sICH among the three stroke categories (23.7%), compared to either Caucasians, African Americans, or ‘Other’ as well as to combined all non-Asians (21.0%). The largest inter-ethnic difference was in the greater proportion of sICH among all stroke types in Asians (23.7%) compared to African Americans (17.7%). Moreover, Asian Americans had the highest apparent incidence of sICH (30/100,000/yr) compared to the other ethnic groups (for example two-folds higher than Caucasians). However, these differences did not reach overall significance compared to either Caucasians alone or combined non-Asians (X2=4.6, p>0.50). Interestingly, the percentage of all strokes that were SAH in Asians (6.5%) was less than in combined non-Asians (8.5%) or in Caucasians alone (8.8%). The low prevalence of SAH in Asians is reflected in the highest Chi Square component analysis score (X2=2.13) compared to sICH, the next highest (X2=1.80).

**Table 2:** Stroke types by ethnicity admitted to neurological service between Jan 2017 and December 2021 Asian Americans include ancestry from far and southeast countries as well as the Indian subcontinent. 'Other' includes Hispanic, Native American/Alaskan and unknown. Hemorrhagic transformations and fibrinolytic hemorrhagic complications of AIS are not included. Primary ICH and SAH categories exclude traumatic brain injuries and isolated epi/subdural hematomas. Top: Case numbers, Middle: percentages of each type of stroke across ethnicity, Bottom: percentage of stroke types within each ethnic background. Although Asians had the highest percentage of ICH among stroke types compared to combined all non-Asians (23.7% vs. 21.0%), Chi Square comparisons were non-significant (Asian vs. non-Asian, X2= 3.5, p>0.5 and Asian vs. Caucasian, X2=4.0, p>0.5), see text. *Apparent Incidence (no./100K/yr) was derived from dividing the number of strokes of a given type admitted to one Boston institution over 5 years by the whole adult population over age 18 within each ethnic group (using 2020 Boston census data). Example calculation: In Asian-Americans, the total strokes over 5 yrs/2020 census is 350/55,752=126/100,000/yr. 126/100K x 83/350 (0.237, the proportion of sICH)=30/100,000/yr. There is an apparent ICH risk ratio (RR) of approximately 2 in Asian Americans relative to Caucasians, see text. Abbreviations: AIS - acute ischemic stroke, ICH - intracranial hemorrhage, SAH -subarachnoid hemorrhage, sICH - spontaneous intracranial hemorrhages.

Race	Stroke type			Total
Counts	AIS	ICH	SAH	total
Asian	244	83	23	350
White	752	231	95	1078
Black/African American	115	27	10	152
Other	81	25	10	116
Total	1192	366	138	1696
Percentage : stroke type	AIS	ICH	SAH	Proportion
Asian	20.47%	22.68%	16.67%	0.206
White	63.09%	63.11%	68.84%	0.635
Black/African American	9.65%	7.38%	7.25%	0.09
Other	6.80%	6.83%	7.24%	0.069
Total	100.00%	100.00%	100.00%	1
Percentage: ethnicity	AIS	ICH	SAH	Total
Asian	69.71%	23.71%	6.57%	100%
White	69.75%	21.43%	8.81%	100%
Black/African American	75.65%	17.76%	6.58%	100%
Other	69.82%	21.55%	8.62%	100%
Incidence: no./100K/yr*	AIS	ICH	SAH	2020 Census
Asian	87.8	30	8.3	55,752
White	50.7	15.5	6.4	296,394
Black/African American	16.7	3.9	1.5	137,673
Other	10.5	3.3	1.3	154,170

## Discussion

We identified six categories of ICH in our Asian American population by broad mechanisms or characteristics, some unusual. These are vasculopathy, hypertensive crises, moyamoya disease/syndrome, venous sinus thrombosis, brainstem hemorrhages and arterial malformations /aneurysm. Our selected case series of less common, but not rare, cases is first notable for a generally young age of onset, less than 65 in 10 out of the 13 (Table 1). They serve as examples to discuss research into mechanisms of ICH risk in an Asian American population.

The vasculopathy cases 1 and 2 both had evidence for MCA disease with vasospasm. It is noted that moderate MCA stenosis, already more prevalent in the Chinese with ischemic stroke (IS), is associated with a greatly increased risk of spontaneous (s)ICH (OR 9.9 to 5.0) in the lenticulostriate territory, possibly related to increased perfusion pressure [17,18]. In this regard, we are unaware as to whether Transcranial Doppler (TCD) MCA velocity measurements are in use to screen Asian patients with a first ischemic stroke or transient ischemic attack (TIA). Although we did not find cases of Cerebral Autosomal dominant Arteriopathy with Sub-cortical Infarcts and Leukoencephalopathy (CADASIL) or the recessive form (CARASIL), it should be mentioned that Asians present with fewer anterior temporal hyperintensities on MRI and less migraine/seizure than Caucasians, but more ICH. Certain exonic mutations in the *NOTCH3 *gene among Asians may account for the differential phenotype [19].

The moyamoya cases 5 and 6 illustrate that ICH can complicate the more usual presentation of IS. Cerebral microbleeds (CMBs) are more frequent and the risk of ICH associated with them is higher in the Asian moyamoya disease/syndrome population than in the USA population as a whole. In non-Asians with moyamoya, the prevalence of CMB is 16% but ICH occurred in none [20]. More generally, CMBs in Asian patients with AFseem to increase the risk of warfarin-associated ICH, and so the use of NOACs should be considered instead of coumadin [21]. The vascular anatomy in Asian patients with moyamoya becomes altered during aging relative to non-Asians, which could lead to the ICH risk. For instance, posterior vascular dilatation of collateral pathways (posterior choroidal artery (pChoA) and posterior communicating artery (PComA)) exceeds that found in the lenticulostriate territory, resulting in a differential reduction of the hemodynamic reserve [22].

The brainstem cases 9 and 10 in young adults with co-existent DM, HTN, and smoking suggest a predisposition to ICH from common metabolic stroke risk factors. Among Chinese cohorts, there is a linear inverse relationship of total cholesterol and HDL cholesterol with ICH (HR 1.43, p=.03) [23,24]. Genetic polymorphisms may be responsible for increased susceptibility to ICH among Asians, for instance, in the *ACE* [25], *RAGE *[26], and *CD36 *[27] genes. Interestingly, the APOE4 genotype was only associated with ICH in white but not Asian population [28]. However, it appears to confer increased risk for aneurysmal SAH and poorer outcome in Asians (OR 4.99) [29,30].

Our venous thrombosis-related parenchymal hemorrhages (cases 7 and 8) were not associated with cerebral venous malformations. Rather these were associated with venous thromboses from platelet and coagulation conditions. Nevertheless, it is suspected that infratentorial cerebral venous malformations are particularly prone to hemorrhage in Asians [31]. Indeed, case 9 could fit this condition. The incidence of familial cerebral cavernous malformations in Asians is unknown.

The hypertensive arteriopathy cases 3 and 4 suggest that a combination of hypertension and arterial wall disease from other causation may predispose Asians to ICH. One of our patients had polycystic kidney disease (PCKD) and a non-aneurysmal ICH. A Japanese population study reporting on gene array findings found that a PCKD-1-like gene polymorphism was associated with SAH [32]. Collagen is important for vessel wall elasticity and polymorphisms in the type 1 collagen gene (*COL1A1* and *2*) may play a role in hypertensive-related ICH among Asians [33,34]. Moreover, genetic variations in the *COL4A1 *gene were also found to be associated with ICH severity in a Chinese population (OR 2.26) [35]. Interestingly, while polycystin gene mutations (*PKD1 *and *2*) cause >90% of autosomal dominant PCKD, a kidney ciliopathy, a similar phenotype was found in a pedigree with a *COL4A1 *mutation [36].

The arterial-venous malformation/aneurysm cases 11 to 13 were severe due either to treatment complications, multicompartmental hemorrhages, infarction, and/or death.

Curiously, we found no case of ICH among our Asian population that was due to Cerebral Amyloid Angiopathy (CAA). Multiple (>2) lobar microhemorrhages, siderosis, and/or macro lobar hemorrhages sparing deep hemispheric or brainstem territories are magnetic resonance (MR) criteria for probable CAA [37,38]. Sporadic CAA usually occurs in more elderly patients, eighth decade or older, and especially in those with dementia. CAA-associated ICH comprises 10-34% of all ICH in Western populations [39] but little is reported specific to Asians, outside Japan [40]. One report among Chinese gives a prevalence of CAA in sICH of only 8.2% [41], while another cites 37.7% [42]. Nevertheless, lobar ICH comprises only 3.4% of all ICH in Asians, compared to 15.4% in Caucasians [43] in agreement with the much lower prevalence of CAA in Asians.

At our institution, situated in a dense, non-affluent Asian community, we found a small increase in the proportion of sICH among all stroke types in Asian patients relative to combined non-Asians, as well as relative to Caucasians alone, however, these did not reach statistical significance. We also found that an ‘apparent’ incidence of sICH in Asian Americans was two times higher than in Caucasians, in general agreement with the aforementioned studies. One of our study’s limitations is that the latter calculations are only ‘apparent’ and not a ‘true’ incidence because the denominator reflects the ethnic adult population census across the whole city, whereas the stroke numbers come from only one of five city-wide certified comprehensive stroke centers. Thus, the absolute number as calculated is an underestimate, a shortcoming that reflects not capturing all ICH in the region. Moreover, it is possible that a larger proportion of the Asian community within the city limits is admitted or referred to our facility for stroke, inflating that number relative to the other ethnic groups. Finally, the increase in proportion and incidence found in this preliminary study did not reach statistical significance so no firm conclusion can be made. Nevertheless, we propose that even these small local and institutional differences lend support to the notion that unknown heritable factors might be innately retained in US Asians so as to affect their ICH risk and therefore deserving more investigation.

The causes of sICH, including SAH and hemorrhagic transformation of ischemic stroke, are complex and involve the interaction of genetic and environmental/lifestyle factors. Where an ethnic group has a significant increase in the OR for sICH, an opportunity presents itself to uncover the genetic markers that predict risk through genomic research. Many candidate vascular genes of interest have been studied for an association with ICH risk in Asian populations. Examples include: *ACE *gene polymorphisms in hemorrhagic stroke (OR 1.97 vs. 1.02 in Caucasians) [25,44]; various inflammatory gene polymorphisms - transforming growth factor-beta​​​​(TGFβ-1), tumor necrosis factor-alpha (TNFα) [45-48], structural gene variants such as the degrading enzyme ​​​​​​​matrix metalloproteinase-9 (MMP-9) and its inhibitor tissue inhibitors of metalloproteinases​​​​​​​-1 (TIMP-1)[49-51]; and others, e.g., endothelin-1 and fibrinogen polymorphisms [52,53]. While these variants each range in OR from 1.5 to 2.0, they individually have a relatively small effect on the risk of complex outcomes such as ICH and cardiovascular disease [1]. Nevertheless, genetic (inherited) variants may account for as high as 30-60% of cardiovascular disease [54] and 1/3 of all ICH risk [55,56]. Similarly, 42% of the variance in ICH incidence is explained by ethnicity [3].

The majority of the aforementioned biomarker publications are candidate gene polymorphisms and allele case-control studies using DNA sequencing and genotyping methods [1]. In order to widen the detection of susceptibility variants, genome-wide association studies (GWAS) are widely used. GWAS uses high throughput genotyping to assay hundreds of thousands of SNPs (single nucleotide polymorphisms), VNTR (variable number tandem repeat), and CNV (copy number variants) to identify risk or protective alleles associated with a condition. For instance, one cross-ethnic GWAS of ruptured and unruptured intracranial aneurysms identified a polygene architecture of 17 SNP risk loci accounting for 21% heritability. This was then conditioned on risk traits that drive the genetic correlation, such as smoking and HTN, to explain over half of total disease heritability [57]. Another large GWAS meta-analyzed several European data sets for intraparenchymal ICH and found two chromosomal susceptibility loci out of 5 million SNPs [58]. However, GWAS probing sICH are lacking in Asian populations. A hypothetical study might combine the data of thousands of individuals within case-control Asian and non-Asian populations in a meta-analysis to yield a panel of SNPs that can later be enriched for traditional risk factors, like HTN, to yield Genetic Risk Scores (GRS) which could improve risk classification for sICH. A limitation of GWAS is that they will not yield functional or mechanistic insight beyond knowing, for instance, that various susceptibility loci are near specific genes for endothelial function. So, these must be tested in endothelial, smooth muscle, and other vascular cell models for marker validation, as for instance in coronary artery disease [59,60] and diabetes [61].

Certain practical considerations arise from this review regarding anticoagulation, thrombolysis, and statin use in Asians with non-valvular AF and prior ICH or CMBs. First, the risk of ICH on warfarin therapy is notably higher in Asians (173 cases/18,867 patients/5 yrs) compared to Caucasians (HR 4.06 [10,62] to HR 2.0-2.4 [63-65]), a finding confirmed in Japanese [66]. Fortunately, NOACs are superior to warfarin both for stroke prophylaxis (HR 0.77) and to lower the risk of recurrent ICH in Asian populations (HR 0.66 and 0.56 [67-69]). Statin use significantly decreased the risk of a first ICH in Asians with hypercholesterolemia (HR=0.78)[70]. Whether statin use, in general, can reduce sICH risk in Asians, as it did regardless of lobar or non-lobar location (OR=0.83) in a large Danish registry study [71], remains to be seen. As for rtPA in AIS, the Taiwanese population was not at higher risk for symptomatic ICH (4.8%) [72], whereas an international study comparing outcomes in Asian and non-Asian populations concluded a higher risk of ICH and earlier deterioration in Asians relative to non-Asians getting rtPA (OR 1.51) [9].

## Conclusions

In this preliminary study restricted to a local institution, we find that community-based Asian Americans possibly carry an increased risk for strokes classified as sICH, similar to their ancestral populations, but to a lesser extent and not reaching statistical significance. The cases presented highlight some unusual factors that, in sum, support the literature that heritable risk factors account for many such cases. Since a conclusion could not be made, we intend a larger metropolitan study, case-control/retrospective in design, across multiple institutions that use the same integrated medical record-keeping system to answer the risk question. If a larger study confirms significant risk, a broad search for polymorphic alleles will be undertaken. The goal would be to develop predictive genetic tests that inform interventions such as screening angiography, aggressive blood pressure reduction, and anticoagulant modification.
